# Giant Cervical Goiter With Posterior Mediastinal Extension

**DOI:** 10.7759/cureus.1450

**Published:** 2017-07-10

**Authors:** Ashraf Zahra, Osama Abdallah, Gamal A Farag

**Affiliations:** 1 Head of Cardiothoracic Surgery Department, Shebin El Kom Teaching Hospital; 2 MD, General Surgery, Shebin El Kom Teaching Hospital; 3 MD, Assistant Professor Cardiothoracic Surgery, Al-azhar University, Damitta Branch

**Keywords:** : cervicomediastinal goiter, transcervical, thoracotomy, sternotomy

## Abstract

Most cervico-mediastinal goiters are situated in the anterior mediastinal compartment, but according to the literature, 10–15 percent of them are located in the posterior mediastinum. Although most anterior mediastinal goiters can be removed by using the transcervical approach, cervico-mediastinal goiters in the posterior mediastinal may require additional extracervical incisions. We report the case of a huge cervico-mediastinal goiter extending from the neck retrotracheally to the posterior mediastinum. Surgical removal is the treatment of choice in such cases. We performed an operation using a transcervical and right posterolateral thoracotomy approach. Histopathological examination confirmed the diagnosis of a large toxic goiter. The patient recovered well and was discharged in one week. While most retrosternal goiters can be resected through a transcervical approach, those extending beyond the aortic arch are better dealt with by either sternotomy or thoracotomy. This report describes the use of transcervical and posterolateral thoracotomy with an excellent postoperative result.

## Introduction

Diseases of the thyroid such as goiters still exist in many endemic parts of the world [1]. The enlargement of the thyroid gland is because of an iodine deficiency in 90 percent of the cases and because of a selenium deficiency in 10 percent of the cases, especially in African countries, such as Sudan, Tanzania, South Africa, and Zaire [2]. Up to 45 percent of goiter patients may have a substernal component in addition to a cervical component [3]. About 75 to 95 percent of retrosternal goiters are situated in the anterior mediastinal compartment. About 90 percent of them can be resected using only the cervical approach. Posterior mediastinal goiters are uncommon, consisting of 10–15 percent of all mediastinal goiters. Although most anterior mediastinal goiters can be removed using the transcervical approach, posterior mediastinal goiters may require additional extracervical incisions.

## Case presentation

**Figure 1:**
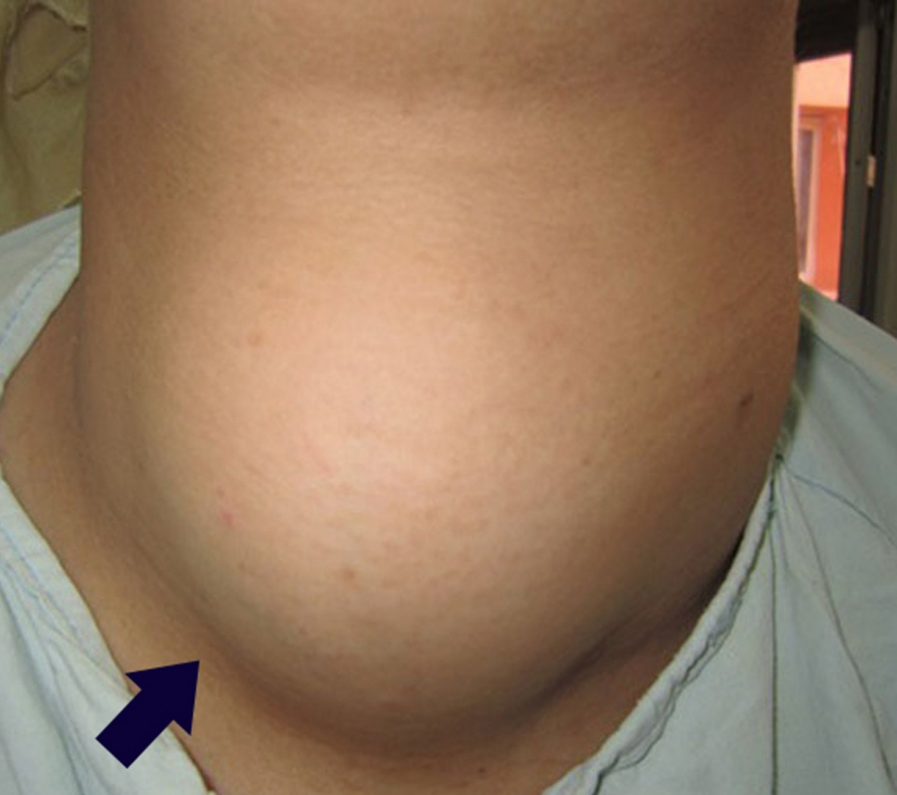
Anterior view

**Figure 2:**
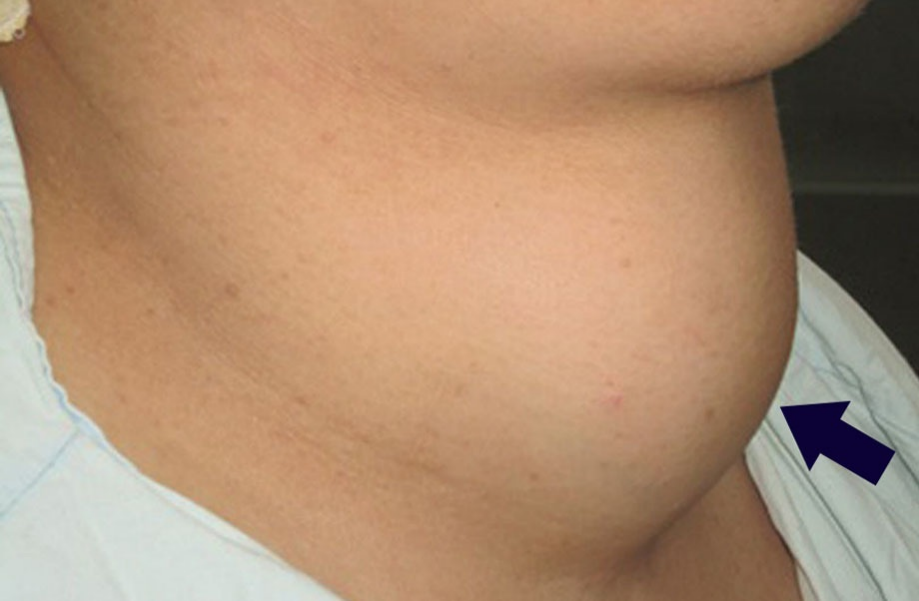
Lateral view

A 39-year-old woman with two children, with a body weight of 125 kg, was admitted to Shebin Elkom Teaching Hospital with a history of cervical swelling that started to appear 13 years ago. There were also vague thoracic symptoms in the form of unspecific chest discomfort. The patient also reported fatigue and dyspnoea during physical stress. A multislice post-contrast computed tomography (CT) scan done in December 2015 revealed that the thyroid gland was noticeably enlarged, with marked heterogeneous enhancement and multiple, bilobar variable-sized nodules or masses reaching superiorly to the level of the floor of the mouth and having a large posterior mediastinal extension into the right hemithorax behind the trachea and the right main bronchus (Figure 1-2). There was a marked attenuation of the laryngeal and tracheal air column with a mild shift to the right side. A noticeable displacement of the posterior mediastinum and carotid sheath vessels was noted with no gross vascular invasion.

**Figure 3:**
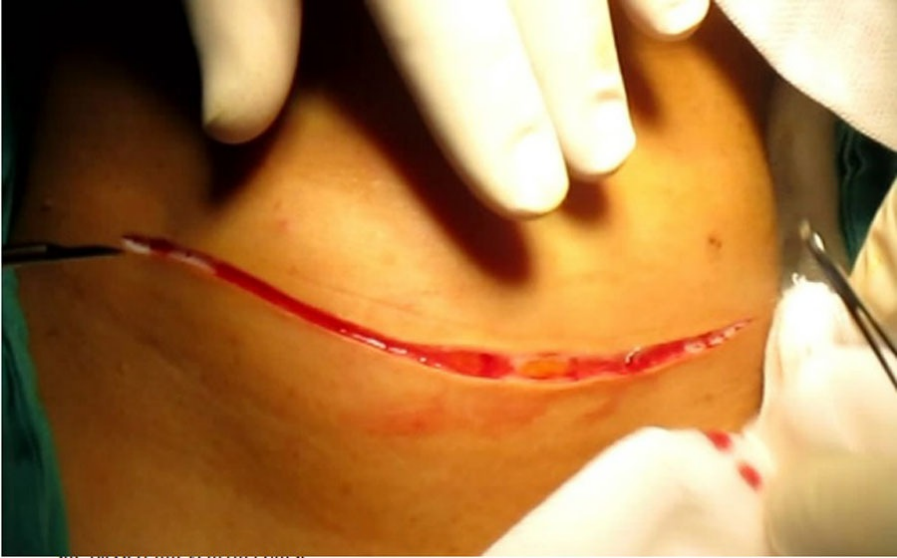
Cervical incision

An ultrasound of the neck revealed that both thyroid lobes and the isthmus were enlarged; all showed multiple variable-sized heterogeneous nodules. The nodules measured about 7x5.5 cm and some nodules showed areas of cystic degeneration. The right lobe = 11x8x7 cm in dimension, the left lobe = 10x7x7 cm in dimension, and the isthmus was 6 cm in anterior-posterior diameter.The patient was admitted to the Shebin Elkom Teaching Hospital and surgery was done by a team of general surgeons, cardiothoracic surgeons, and ear, nose, and throat (ENT) surgeons. A decision was taken to make both cervical and posterolateral thoracotomy incisions to prevent the risk of any future illnesses or death resulting from acute airway obstruction; the patient consented and was prepared for the operation. We performed the operation using the transcervical and right poster-thoracotomy approach. First, we made a collar (Kocher's) incision on the neck (Figure 3).

**Figure 4:**
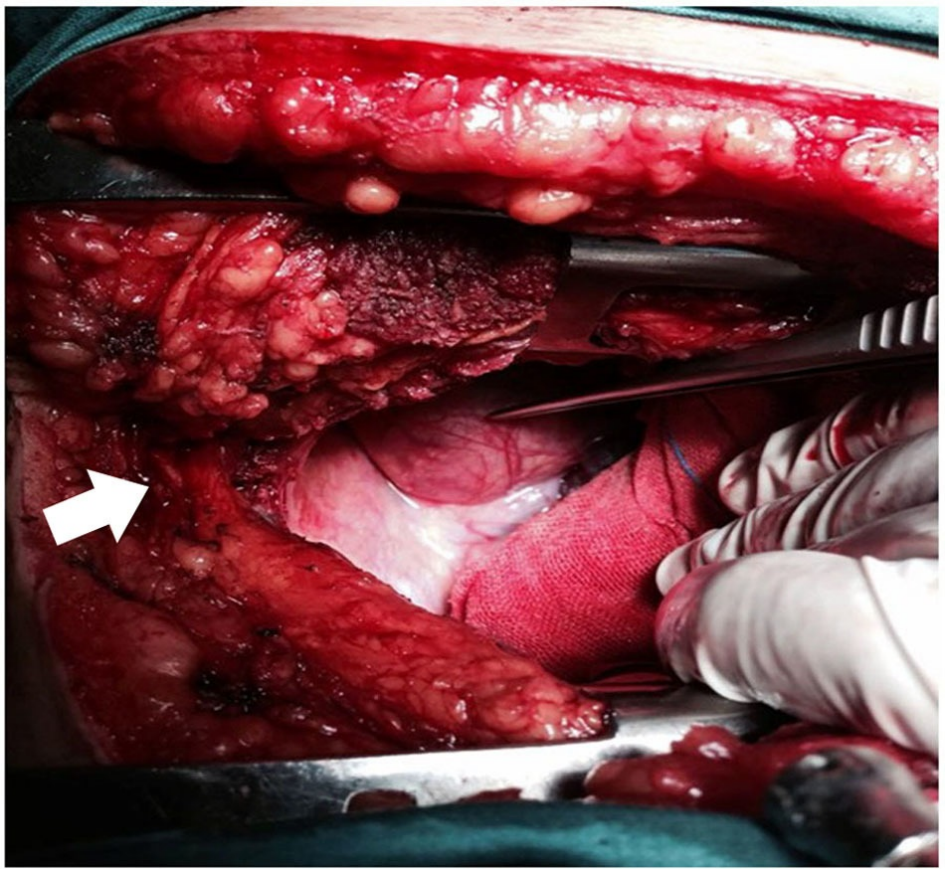
Space between the right lobe of the thyroid gland and the sternum

**Figure 5:**
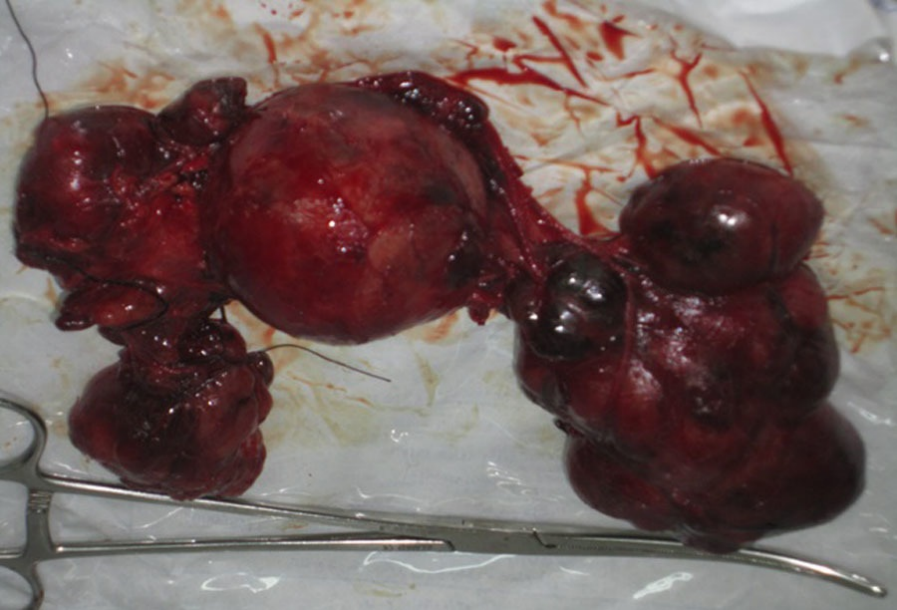
Cervical specimen

We excised the left lobe of the thyroid gland and the isthmus. Then, we excised the cervical part of the right lobe. The superior and inferior thyroid arteries and veins were ligated. During the operation, we had some problems with separating the lower thyroid artery because of the huge mass of the thyroid gland. The separation of the lower thyroid artery was difficult because of the absence of enough space between the right lobe of the thyroid gland and the sternum due to continuity into the mediastinum (Figure 4). Consequently, we slightly elevated the sternum. This allowed the ligation of the inferior thyroid artery. The patient was converted to the left lateral position and a right posterolateral thoracotomy through the bed of the right fourth intercostal space was made. The posterior mediastinum was inspected; it was a large mass extending from the root of the neck down to the azygos arch encroaching on the superior vena cava, the trachea, the right main bronchus medially, and the esophagus and the posterior chest wall posteriorly. It was a pedicle mass reaching up to the right subclavian artery. The dissection was made very cautiously so as not to injure any of the surrounding structures and the mass was extracted (Figure 5).

There was a feeding vessel coming from the right fourth posterior intercostal artery, which was ligated. The chest was closed on two chest tubes. After closing the chest and repositioning the patient, the vocal cords were inspected; they were moving well. Then, the patient was sent to the intensive care unit (ICU) for overnight observation and then to the ward, where she passed an uneventful course of treatment.

## Discussion

There are two known types of mediastinal goiters. The first is the primary type, which is a congenital anomaly of the thyroid tissue lying primarily in the chest and constituting one percent of the types of goiters [4]. The other type is the secondary acquired retrosternal extension. Most substernal goiters lie in the anterior mediastinum; 90 percent of these can be accessed via the collar incision. While attempts at the resection of the posterior mediastinal goiter are challenging, the need for sternotomy or thoracotomy is uncommon [5]. The posterior mediastinal extension is usually right-sided because of the presence of the aortic arch and pulmonary vessels on the left side obstructing the path of descent [6]. This patient surprisingly was asymptomatic with reference to compression manifestations on the airways of the esophagus or superior vena cava. Her symptoms were only related to a long history of a hyperthyroid state and were controlled by medications [7]. In Madjar’s series of 222 thyroidectomies cases over a 22-year period, 40 patients had intrathoracic goiters while only 4 patients required partial sternotomy and 2 required posterolateral thoracotomy. In DeAndrade’s series of 128 substernal goiters with posterior mediastinal extensions, 122 were excised through a transcervical approach; 6 patients, however, required sternotomy and thoracotomy [8]. There is a case report by Sanaz Harirchian in which they could not approach the mass via sternotomy only and this necessitated doing thoracotomy [9].

## Conclusions

Diseases of the thyroid gland, such as goiters, still exist in many endemic parts of the world. We presented the case of a 39-year-old lady with cervical goiter and posterior mediastinal extension, which occurs in only 10 percent to 15 percent of goiters with intrathoracic extension. The mass was receiving separate blood supply from the right fouth posterior intercostal artery. In this case, we conclude that a combined approach using both cervical and posterolateral thoracotomy incisions is sometimes needed. Further, thoracoscopic removal of the posterior mediastinal extension mass is also possible.
